# HIV Risk Perception Among Migrant Girl Head Porters (*Kayayei*) in Ghana

**DOI:** 10.1002/puh2.70330

**Published:** 2026-08-05

**Authors:** Sylvester Kyei‐Gyamfi

**Affiliations:** ^1^ Department of Children Ministry of Gender, Children and Social Protection Accra Ghana

**Keywords:** AIDS perception, Ghana, health belief model, internal migrants, *Kayayei*

## Abstract

This study investigates perceptions and fears of acquired immunodeficiency syndrome (AIDS) among female migrant head porters (locally known as *Kayayei*) in Accra's Makola and Agbogbloshie markets. The term ‘head porter’ refers to young migrant women who transport goods on their heads for a fee in large urban markets, a labour practice common in West African trading environments. Using the health belief model (HBM), the study explores cognitive and emotional responses to human immunodeficiency virus (HIV)/AIDS. A mixed‐methods approach was utilized, including a cross‐sectional survey of 400 respondents, two focus group discussions with 24 participants, and 20 key informant interviews with market leaders and child protection officers. Quantitative results indicated that 83.5% of respondents viewed AIDS as a dangerous disease, whereas 85.5% expressed fear of contracting the infection. Age and education showed no significant association with fear, although junior high school education correlated with greater awareness of disease severity. Qualitative findings identified five major reasons underlying fear, including perceptions of incurability and visible suffering associated with AIDS, whereas those who expressed little fear cited divine protection, confidence in modern treatment or fatalistic beliefs about illness. The HBM illuminated variations in perceived susceptibility, severity and self‐efficacy. Despite relatively high awareness of HIV/AIDS, structural and economic barriers continue to limit the adoption of protective behaviours. The study advocates for culturally responsive HIV interventions that strengthen self‐efficacy, address misconceptions and better reflect the lived realities of migrant adolescent girls and young women.

## Graphical abstract

This study examines HIV/AIDS risk perception among migrant girl head porters (Kayayei) in Accra, Ghana. Although most perceive AIDS as dangerous and fear infection, cultural beliefs, economic pressures and structural barriers shape their responses. Findings highlight the need for culturally responsive HIV interventions that strengthen self‐efficacy and improve access to prevention services.

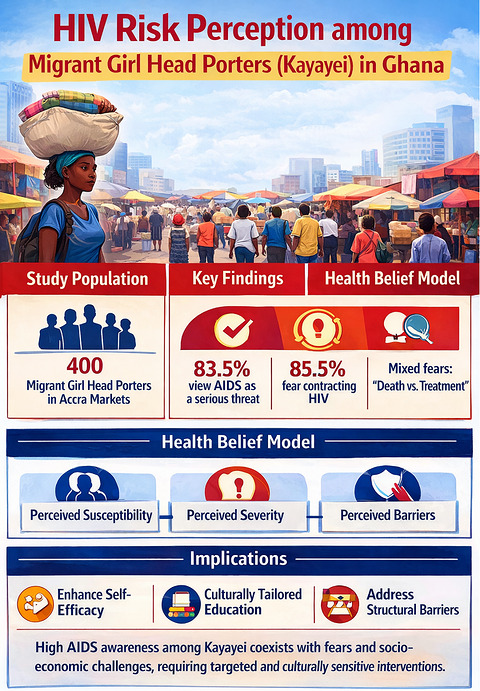

## 1 Introduction

Mobile populations worldwide are frequently identified as groups facing heightened vulnerability to human immunodeficiency virus (HIV) and other sexually transmitted infections (STIs) due to the socio‐economic constraints and social dislocation associated with migration. Populations commonly recognized as high‐risk include fishers, long‐distance drivers, military personnel, sex workers, traders, seasonal agricultural workers, refugees and internal migrants [1, 2]. Their risk profiles are shaped by intersecting structural factors, including physical separation from family support systems, unstable housing conditions, restricted access to health services and the adoption of coping strategies such as transactional sex for economic survival. These mobility patterns often weaken social protection networks and disrupt consistent access to sexual health information and prevention services [3, 4]. Moreover, persistent economic insecurity, limited educational opportunities and social marginalization further amplify the vulnerability of these groups to HIV and other STIs [5]. Consequently, mobile populations have become a key focus of HIV prevention initiatives across sub‐Saharan Africa (SSA) [2, 6].

In Ghana, one of the most marginalized groups of internal migrants is the *Kayayei* (*singular: Kayayoo*), referring to young female head porters who migrate from the northern regions of the country to major southern cities, such as Accra, Kumasi and Takoradi, in search of economic opportunities. The term derives from the Hausa word ‘*kaya’* (goods or load) and the Ga word ‘*yoo’* (woman), reflecting their primary role of transporting goods on their heads within busy market centres [7]. These girls and young women typically fall between the ages of 12 and 25 and often have limited formal education, weak social protection networks and constrained access to health services.

Previous studies have documented multiple vulnerabilities experienced by *Kayayei*, including exposure to sexual exploitation, violence and poor health conditions [8, 9, 10]. Many live in overcrowded informal settlements or temporary shelters near market spaces, where insecurity, poor sanitation and limited privacy are common. Their precarious economic circumstances and social marginalization may compel some to engage in survival strategies, such as transactional sex in exchange for money, accommodation or protection, thereby increasing their vulnerability to HIV and other STIs [8].

Over the past two decades, several HIV prevention and reproductive health initiatives targeting vulnerable urban populations, including migrant young women, have been implemented in Ghana [11, 12, 13]. These include peer education programmes, community outreach campaigns, voluntary counselling and testing services, and youth‐focused sexual and reproductive health education delivered through governmental and non‐governmental organizations [14, 15, 16]. Despite these efforts, evidence suggests that many such campaigns have struggled to achieve sustained behavioural change among marginalized populations, partly because interventions are often insufficiently tailored to the sociocultural realities of culturally and linguistically diverse groups [17].

Although several studies have examined the socio‐economic conditions, migration dynamics and general health vulnerabilities of *Kayayei* [8, 9, 10], relatively few empirical studies have specifically investigated how this population interprets and internalizes HIV/acquired immunodeficiency syndrome (AIDS) risk. Existing research has largely focused on structural vulnerabilities and livelihood challenges rather than the cognitive and emotional perceptions that shape sexual health decision‐making [18, 19, 20].

It is frequently assumed that prolonged exposure to HIV awareness campaigns has improved knowledge and reduced risky sexual behaviour among high‐risk populations [21, 22]. However, empirical evidence from parts of SSA suggests that awareness does not always translate into behavioural change, particularly where structural constraints and cultural interpretations of illness influence decision‐making [2, 23]. Consequently, it remains unclear whether HIV prevention messaging has meaningfully shaped how migrant adolescent girls and young women perceive the risks associated with AIDS.

Understanding how *Kayayei* interpret HIV risk is particularly important given Ghana's current HIV epidemiological profile. National adult HIV prevalence remains relatively low at approximately 1.6%, yet adolescent girls and young women continue to experience disproportionate vulnerability compared to their male counterparts [24]. Urban informal economies, including large market environments, create contexts where young migrant women may encounter heightened exposure to exploitative sexual relationships and limited access to protective health services [20, 25].

This study therefore explores the perceptions, fears and explanatory beliefs that shape how *Kayayei* in Accra understand and interpret the risks associated with HIV/AIDS. The research seeks to determine whether *Kayayei* perceive AIDS as a serious threat, identify factors influencing these perceptions, assess their level of fear regarding infection and explore the reasons underlying both fear and indifference.

Examining these perceptions is important because health behaviour is strongly shaped by individuals’ subjective interpretations of risk. The health belief model (HBM) suggests that preventive behaviours are more likely to occur when individuals perceive themselves as susceptible to a serious health condition and believe that protective actions are both effective and feasible [26]. Understanding the cognitive frameworks through which *Kayayei* interpret HIV risk may, therefore, provide valuable insights for improving prevention strategies.

Furthermore, recent public health literature has noted emerging concerns about ‘HIV prevention fatigue’ in some African contexts, where prolonged exposure to HIV messaging and the growing availability of antiretroviral therapy may reduce the perceived severity of the disease among certain populations [2, 27, 28]. Within this broader context, this study explores both fear and nonchalance toward AIDS among migrant *Kayayei* in Accra, offering insights that may inform more culturally responsive HIV prevention programmes for highly mobile and economically marginalized young women.

## 2 Theoretical Framework: HBM

This study is based on the HBM, a psychological framework developed in the 1950s by social psychologists Hochbaum, Rosenstock and Kegels to explain and predict health‐related behaviours. The model was initially designed to understand why individuals failed to adopt preventive health services, and it has since been widely applied in public health research examining behavioural responses to diseases such as HIV/AIDS [29]. The theory proposes that individuals’ engagement in health‐promoting behaviours, such as condom use or avoidance of unprotected sex, is influenced by several belief‐based constructs related to perceived health threats and the perceived value of preventive actions. These constructs include perceived susceptibility (the perceived likelihood of contracting a disease), perceived severity (beliefs about the seriousness of the disease and its consequences), perceived benefits (beliefs about the effectiveness of preventive actions), perceived barriers (perceived costs or obstacles to taking action), cues to action (triggers that prompt behaviour change) and self‐efficacy (confidence in one's ability to successfully perform preventive behaviour) [30].

The relevance of the HBM to this study lies in its emphasis on how individuals interpret the seriousness of a disease and their personal vulnerability to it. These dimensions align directly with the objectives of this research, which seek to determine whether *Kayayei* perceive AIDS as a serious threat and whether they fear contracting the disease. Understanding these perceptions is particularly important for migrant young women, such as Kayayei, whose health behaviours are shaped by a combination of personal beliefs, social norms and structural constraints within the urban informal economy [23, 31].

Although the HBM was originally developed in Western public health contexts, it has been widely applied in HIV‐related studies across SSA, including research on condom use, HIV testing and treatment adherence. Studies conducted in Ghana and other African settings have demonstrated that the model's constructs, particularly perceived susceptibility, perceived severity and perceived barriers, remain useful for explaining variations in HIV preventive behaviours [32, 33]. However, scholars have also noted that health beliefs in African contexts are often shaped by broader cultural and social influences, including religious interpretations of illness, fatalistic beliefs about disease and community norms regarding sexuality and gender relations [34, 35].

In Ghana, religious and spiritual worldviews frequently influence perceptions of illness and health risk. Illness may sometimes be interpreted through spiritual or moral frameworks, whereas beliefs in divine protection or destiny can shape individual perceptions of vulnerability to diseases such as HIV/AIDS [35, 36]. These cultural interpretations can interact with biomedical understandings of HIV and may influence how individuals respond to prevention messaging [23, 34]. Recognizing these contextual dynamics is therefore important when applying cognitive behavioural models such as the HBM in the Ghanaian context.

Furthermore, the population examined in this study, migrant girl head porters (*Kayayei*), largely originates from the northern regions of Ghana, which are culturally diverse and composed of multiple ethnic groups, including Dagomba, Mamprusi, Gonja, Frafra, and others (Ministry of Gender, Children and Social Protection [MoGCSP] [37]). Although these groups share certain broad regional histories and migration patterns, they also maintain distinct languages, social traditions and cultural practices [37]. Consequently, health beliefs among *Kayayei* may reflect both shared regional experiences and culturally specific interpretations of illness, morality and vulnerability. Recognizing this diversity is important when applying behavioural models, such as the HBM, as perceptions of disease risk may be shaped not only by individual cognition but also by collective cultural narratives and social environments.

The HBM provides a useful framework for examining how objective knowledge and subjective perceptions of risk interact to influence health decisions. Fundamentally, the model helps explain how individuals cognitively interpret health threats within their everyday social realities. For *Kayayei*, these interpretations are likely shaped not only by health education messages but also by migration experiences, economic pressures and social marginalization within urban market environments.

One of the main strengths of the HBM is its predictive usefulness in public health research. The framework has been widely used in studies examining HIV prevention behaviours, helping to explain why some individuals adopt protective measures, whereas others do not [38]. Its conceptual simplicity and flexibility make it suitable for both qualitative and quantitative studies, particularly those seeking to understand health perceptions among vulnerable or mobile populations. The model's constructs offer a structured yet adaptable framework for analysing the cognitive and emotional dimensions of health‐related decision‐making.

Nevertheless, the HBM has certain limitations. Critics argue that the model focuses primarily on individual cognition and may insufficiently account for the structural, cultural and socio‐economic factors that shape health behaviour [26, 39]. In the case of Kayayei, broader structural conditions, including poverty, gender inequality, insecure housing and exposure to transactional sexual relationships, can significantly influence behaviour regardless of individual perceptions of risk [10, 31]. Furthermore, the model assumes that individuals make relatively rational health decisions based on perceived risks and benefits [30, 39]. However, in contexts where survival needs are urgent, individuals may knowingly engage in risky behaviours despite awareness of potential health consequences [23, 40].

Additionally, in many Ghanaian contexts, religious beliefs, community norms and moral interpretations of illness may influence how individuals interpret susceptibility to diseases such as HIV/AIDS. For example, some individuals may associate illness with spiritual causes, divine protection or moral behaviour, which may shape perceptions of risk independently of biomedical knowledge [35, 41]. These sociocultural influences do not invalidate the HBM but rather highlight the importance of interpreting its constructs within the broader cultural and social environments in which individuals live.

Despite these limitations, the HBM remains a useful analytical framework for this study because it provides a systematic approach to examining perceptions of AIDS risk and fear among *Kayayei*. In this research, the model is applied with an awareness of the broader sociocultural and economic context shaping the lives of migrant head porters. Rather than treating health behaviour as purely individual decision‐making, the framework is interpreted alongside structural realities such as migration, economic vulnerability and social marginalization.

In conclusion, the HBM offers a valuable conceptual lens for analysing how *Kayayei* perceive the threat of AIDS and how these perceptions influence their attitudes and fears regarding infection. The model informs the design of the study instruments and guides the interpretation of findings related to perceived susceptibility, severity and behavioural motivations. By situating the HBM within the Ghanaian sociocultural context, this study contributes to a more context‐sensitive understanding of health beliefs among migrant young women and provides insights for designing HIV interventions that resonate with the lived experiences of *Kayayei* in urban Ghana.

## 3 Methods

### 3.1 Study Setting

This research was conducted in Makola market and Agbogbloshie, two prominent commercial centres within the Accra Metropolitan District in Ghana's Greater Accra Region. These sites were purposively selected due to their high concentrations of *Kayayei*, or female head porters, who are predominantly young migrants from Ghana's northern regions in search of economic opportunities in urban areas [31, 42].

Although many *Kayayei* migrate primarily for economic reasons, some younger migrants also attempt to continue schooling while working in urban markets. In this study, the reference to ‘school‐going migrants’ refers to young migrants who combine income‐generating activities with intermittent or past participation in formal schooling rather than a distinct category of migrants. These individuals remain economically active as head porters while maintaining varying degrees of engagement with education.

### 3.2 Study Design and Data Collection

The study employed a cross‐sectional design, utilizing secondary data extracted from a broader mixed‐methods dataset compiled by the Department of Children (DOC) under Ghana's MoGCSP. The original data collection was conducted by trained researchers and field officers affiliated with the DOC, not by the author of this article. Although the overarching aim of the original dataset was to investigate the living and working conditions of migrant children in urban Ghana, the current article draws specifically on components of the data that focus on *Kayayei*’s perceptions of AIDS danger and fear.

This article limits its focus to four core questions aligned with its objectives: whether *Kayayei* perceive AIDS as a dangerous disease, their reasons for that perception, whether they are afraid of AIDS, and why or why not. These issues were examined using structured questionnaire items and qualitative responses obtained through interviews and focus group discussions (FGDs). Participants were recruited through a combination of convenience and snowball sampling, targeting female migrants aged 10–17 actively engaged in head porterage.

Individuals aged 18 years and above were not included because the original study from which the dataset was derived was designed primarily to investigate the living and working conditions of migrant children. Consequently, the sampling frame focused on children and adolescents under the age of 18. The present study therefore retains this age range to remain consistent with the design and ethical parameters of the original dataset.

**FIGURE 1:**
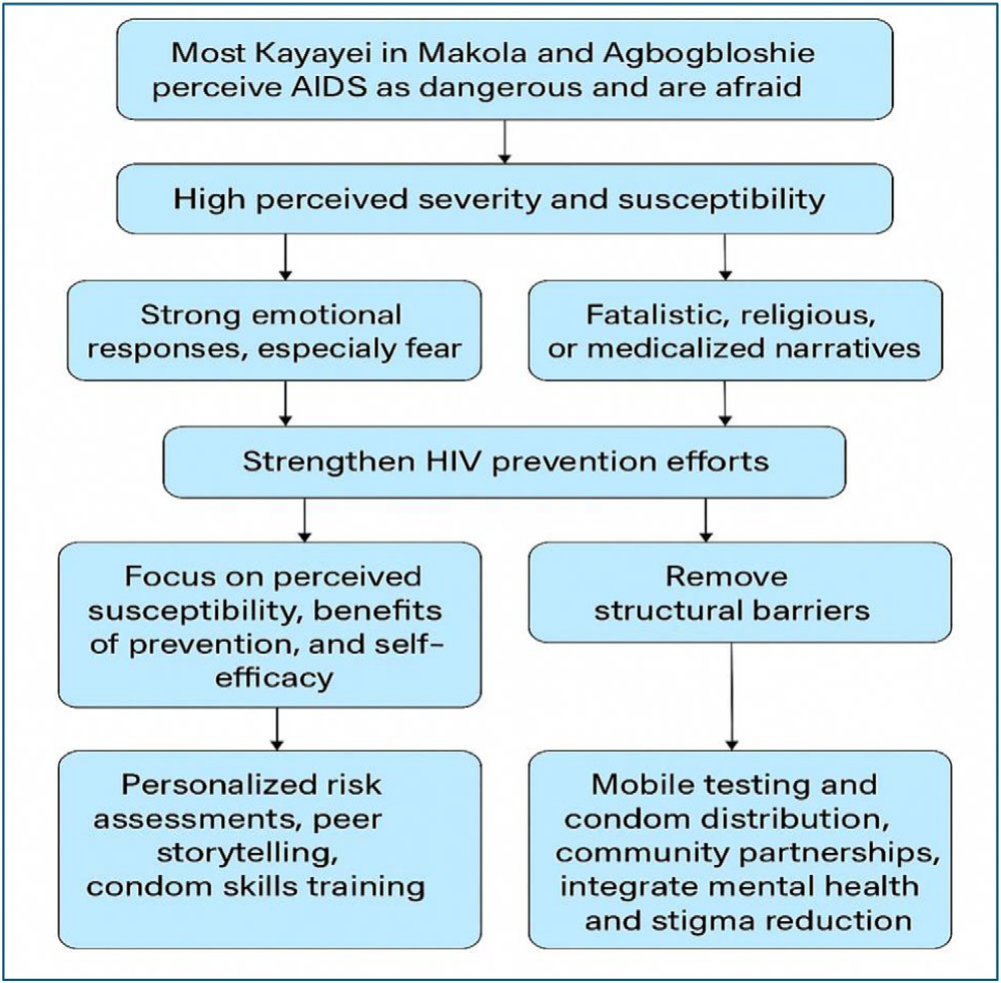
Conclusion and implications for policy and practice. AIDS, acquired immunodeficiency syndrome; HIV, human immunodeficiency virus.

Males, non‐migrants, and individuals aged 18 years and older were excluded. This article focuses exclusively on the responses of *Kayayei* that relate to their perceptions and feelings about HIV/AIDS. A conceptual framework (see Figure 1) guided the methodological approach, detailing the logical flow from site and participant selection through to data collection and analysis.

### 3.3 Data Collection Procedures

The dataset comprised 400 completed survey questionnaires, two FGDs with 24 participants (12 per group), and 20 key informant interviews (KIIs) involving market leaders, representatives from *Kayayei* associations and child protection officers. Migrant peers and gatekeepers were not included as primary analytical units in this study because the original dataset focused specifically on migrant children engaged in head porterage. However, market leaders, association representatives and child protection officers were interviewed as key informants to provide contextual insights into the living and working conditions of *Kayayei*.

For the purposes of this study, attention was paid to survey and qualitative items that captured participants’ beliefs about the danger of AIDS, their levels of fear toward the disease and the reasons given for those beliefs. FGDs and KIIs were guided by semi‐structured interview protocols that included specific questions on knowledge of HIV/AIDS, emotional responses to it and perceived vulnerability or immunity.

To accommodate linguistic diversity and promote effective communication, interviews were conducted in English, Twi, Dagbani and other northern Ghanaian dialects. Language interpretation was provided during data collection by trained interpreters employed by the DOC to facilitate communication between researchers and participants who spoke different local languages. Sessions lasted between 30 and 60 min and were held in locations chosen by participants to ensure privacy and comfort. All discussions were audio‐recorded with informed consent, and complementary field notes were also taken.

After data collection, audio recordings were transcribed and translated into English for analysis. The use of trained interpreters during interviews and standardized transcription procedures helped enhance the credibility and consistency of the qualitative data.

Key domains explored in this article included participants’ personal fear of AIDS, beliefs about the seriousness of the disease and narratives explaining why some are more or less afraid of it.

### 3.4 Measures

Several key variables were measured in this study using a structured questionnaire administered to *Kayayei* respondents. Participants’ age was collected in completed years and later grouped into three categories: 10–12, 13–15 and 16–17 years. The market area of operation was assessed by asking participants to indicate where they worked, with responses limited to the two selected sites, Makola market and Agbogbloshie.

Educational attainment was measured by asking respondents about the highest level of formal education they had completed. Responses were categorized into three levels: never been to school, primary education and junior high school (JHS).

To assess perceptions of HIV/AIDS, participants were asked whether they considered AIDS to be a dangerous disease. Responses to this question were captured as ‘Yes’, ‘No’ or ‘Don't Know’. This was followed by an open‐ended question prompting participants to explain their reasons for perceiving AIDS as either dangerous or not dangerous. The responses were recorded in the participants’ own words and later grouped into thematic categories.

Additionally, the study measured fear of AIDS by asking participants whether they were afraid of the disease. This was captured using a simple binary response format, ‘Yes’ or ‘No’. Reasons for perceiving AIDS as dangerous or not dangerous were measured through an open‐ended follow‐up question asking participants to explain their response to the danger perception question. Responses were recorded verbatim and later categorized during analysis.

### 3.5 Data Analysis

The qualitative data were transcribed verbatim and analysed thematically using NVivo software (version 12). The analysis was guided by a thematic analysis framework consistent with the principles outlined by Braun and Clarke [43]. An inductive analytic approach was employed to identify emerging themes relevant to the study's objectives. A codebook was developed to guide the analysis, and two researchers independently coded the data to enhance inter‐coder reliability. Themes of interest included expressions of fear, perceived severity of AIDS, religious or cultural interpretations and experiences or knowledge shaping these perceptions.

Quantitative data were analysed using the Statistical Package for the Social Sciences (SPSS), version 25. Descriptive statistics were used to summarize demographic characteristics such as age, education level, market area of operation and region of origin. Cross‐tabulations were conducted to explore associations between participants’ perception of AIDS danger and their demographic backgrounds. The integration of qualitative and quantitative data enabled triangulation of findings, offering a nuanced understanding of how *Kayayei* conceptualize the threat of HIV/AIDS.

### 3.6 Ethical Considerations

This study draws on secondary data derived from a broader field exercise conducted by the DOC under MoGCSP. The original data collection formed part of a routine governmental exercise undertaken by the DOC to gather information on the living and working conditions of migrant children in urban markets. The purpose of this exercise was to generate evidence to support Ghana's periodic reporting obligations to the United Nations Committee on the Rights of the Child (CRC), as required under the UN Convention on the Rights of the Child [44].

Because the study formed part of an official governmental data‐gathering exercise rather than an independent academic research project, separate ethical approval from a university ethics review board was not sought. However, the study methods and data collection tools were formally reviewed and approved by Ghana's National Child Protection Committee (NCPC), the statutory national body responsible for overseeing policies and interventions related to child protection. Following this review, the committee granted authorization in February 2020 for the commencement of the fieldwork in April 2020 (Approval Reference: DC/CPC/156). The NCPC assessed the study methodology and data collection instruments prior to implementation to ensure compliance with national child protection standards and internationally recognized ethical principles governing research involving minors. Accordingly, the fieldwork was conducted in line with national child protection guidelines and established ethical standards for research involving children ([44, 45, 46]).

Because participants were minors aged 10–17 years, additional child protection measures were implemented during data collection. Participants were informed about the purpose of the exercise and their voluntary participation. Verbal assent was obtained from all participating children after the study procedures were explained in languages they understood, consistent with established ethical guidelines for research involving children and adolescents [45, 47]. For participants who were accompanied by guardians or responsible adults, guardian consent was obtained before participation.

In situations where migrant children were living independently and no immediate guardian was present, a common situation among Kayayei, trained child protection officers from the DOC, supervised the data collection process and ensured that participation complied with national child protection standards. These officers acted in a protective oversight role to safeguard the rights and welfare of participating children, a practice recommended in research involving unaccompanied or vulnerable minors [45, 48].

All interviews and discussions were conducted in settings chosen by participants to ensure privacy and comfort. Interviewers were trained to recognize signs of distress and to respond appropriately to disclosures of abuse or exploitation. Where such concerns arose, participants could be referred to appropriate child protection services coordinated through the DOC and associated social welfare agencies, consistent with recommended ethical protocols for safeguarding vulnerable child participants in research [45, 48].

Participants were assured that their responses would remain confidential and that no personally identifiable information would be reported. For participants with limited literacy, the consent and assent procedures were explained verbally to ensure full understanding before participation, in line with ethical guidelines emphasizing inclusive consent procedures in research with vulnerable populations [47].

Finally, the present study analyses anonymized data extracted from the original dataset. The consent procedures of the original exercise permitted the use of de‐identified information for further analysis aimed at improving child protection policy and programming. The use of anonymized secondary data for research purposes is widely recognized as ethically acceptable when participant confidentiality is protected and the original consent procedures permit such use [49, 50]. No identifying information about participants is included in this article.

## 4 Results

### 4.1 Socio‐Demographic Characteristics

The socio‐demographic characteristics of the *Kayayei* were assessed, revealing several key insights. Table 1 indicates that among the participants, the majority were aged between 16 and 17 years, comprising 54.8% of the total sample. This was followed by those aged 13–15 years, who represented 23.8%, and younger participants aged 10–12 years, accounting for 21.5%. In terms of the market areas where the *Kayayei* operated, a significant proportion worked in Agbogbloshie, making up 65.0% of the sample, whereas 35.0% were based in Makola market.

**TABLE 1:** Socio‐demographic characteristics of respondents.

Variables	Frequency	Per cent
**Age**		
10–12	86	21.5
13–15	95	23.8
16–17	219	54.8
**Market *Kayayei* operate**		
Makola market	140	35.0
Agbogbloshie	260	65.0
**Educational attainment**		
Never been to school	93	23.3
Primary	267	66.8
Junior secondary school	40	10.0
**Total**	400	100

Regarding educational attainment, the findings showed that a substantial number of participants had not received any formal education, with 23.3% reporting that they had never been to school. The majority, 66.8%, had completed primary education, whereas only 10.0% had attained JHS education as seen in Table 1.

### 4.2 Whether *Kayayei* Perceive AIDS as a Dangerous Disease or Not

Table 2 presents the responses of *Kayayei* on whether they perceive AIDS as a dangerous disease, disaggregated by age group and educational level. Out of the total respondents, 83.5% (*n* = 334) affirmed that AIDS is a dangerous disease, whereas 13.0% (*n* = 52) stated it is not, and 3.5% (*n* = 14) indicated they did not know.

**TABLE 2:** Perceptions of acquired immunodeficiency syndrome (AIDS) as a dangerous disease by age group and educational level.

	Whether AIDS is a dangerous disease?			
Variable	Yes (%)	No (%)	Don't Know (%)	*p* value
**Age**				0.256
10–12	71 (82.6)	12 (14.0)	3 (3.5)	
13–15	81 (85.3)	14 (14.7)	0 (0.0)	
16–17	182 (83.1)	26 (11.9)	11 (5.0)	
**Education**				0.390
Never been to school	77 (82.8)	11 (11.8)	5 (5.4)	
Primary	220 (82.4)	38 (14.2)	9 (3.4)	
JHS	37 (92.5)	3 (7.5)	0 (0.0)	
**Total**	334 (83.5)	52 (13.0)	14 (3.5)	

Abbreviation: JHS, junior high school.

Across age groups, those aged 10–12 years showed an 82.6% agreement that AIDS is a dangerous disease, with 14.0% responding ‘No’ and 3.5% indicating ‘Don't Know’. Among the 13–15‐year‐olds, 85.3% perceived AIDS as dangerous, 14.7% said it was not dangerous, and none reported uncertainty. For the 16–17‐year age group, 83.1% viewed AIDS as dangerous, 11.9% said it was not, and 5.0% responded ‘Don't Know’. The *p* value for the association between age and perception was 0.256.

When categorized by educational level, 82.8% of participants who had never been to school believed AIDS is a dangerous disease, 11.8% did not, and 5.4% were unsure. Among those with a primary education, 82.4% perceived AIDS as dangerous, 14.2% did not, and 3.4% responded ‘Don't Know’. Among respondents who had completed JHS, 92.5% perceived AIDS as dangerous, and 7.5% did not, with no participant expressing uncertainty. The *p* value for the association between educational level and perception was 0.390.

### 4.3 Reasons *Kayayei* Perceive AIDS Is a Dangerous or Non‐Dangerous Disease

Table 3 presents the reasons given by the migrant girl head porters (*Kayayei*) for perceiving AIDS either as a dangerous or non‐dangerous disease. A total of 386 responses were recorded. The difference between the total sample size (*n* = 400) and the number of responses presented in Table 3 (*n* = 386) reflects instances where some respondents did not provide a reason for their response or where the responses were incomplete and therefore could not be categorized during analysis.

**TABLE 3:** Stated reasons for perceiving acquired immunodeficiency syndrome (AIDS) as a dangerous or non‐dangerous disease.

Response	Frequency	Per cent
It kills fast	152	39.4
It has no cure	92	23.8
Claims more lives than any disease	37	9.6
Attacks unfaithful and careless people	35	9.1
Awareness campaigns have reduced its danger	4	1.0
Causes premature deaths	18	4.7
Weakens the immune system	31	8.0
Only affects cursed people	17	4.4
Total	386	100.0

The most frequently cited reason was the belief that AIDS ‘kills fast’, accounting for 152 responses or 39.4 per cent of the total. This was followed by the perception that the disease ‘has no cure’, mentioned by 92 respondents, representing 23.8 per cent. Another 9.6 per cent (*n* = 37) of the *Kayayei* indicated that AIDS ‘claims more lives than any other disease’, whereas 35 respondents (9.1%) attributed the danger of AIDS to the idea that it ‘attacks unfaithful and careless people’. Additionally, 31 responses (8.0%) noted that AIDS ‘weakens the immune system’, and 18 participants (4.7%) highlighted its role in causing ‘premature deaths’. A smaller group of respondents (*n* = 17 or 4.4%) believed that AIDS ‘only affects cursed people’. Meanwhile, four responses (1.0%) suggested that ‘awareness campaigns have reduced its danger’, indicating a minor perception that the disease may no longer be as threatening due to public education efforts.

### 4.4 Whether *Kayayei* Are Afraid of AIDS?

Table 4 displays the distribution of *Kayayei* who reported being afraid of AIDS, analysed by age group and level of education. Overall, 85.5% (*n* = 342) of respondents indicated that they are afraid of AIDS, whereas 14.5% (*n* = 58) stated that they are not. By age group, 86.0% of those aged 10–12 reported fear of AIDS, compared to 84.2% of those aged 13–15 and 85.8% of those aged 16–17. Correspondingly, 14.0%, 15.8% and 14.2% in the respective age groups reported not being afraid. The association between age group and fear of AIDS showed a *p* value of 0.013, indicating a statistically significant association between age group and fear of AIDS. Regarding educational level, 87.1% of *Kayayei* who had never attended school reported being afraid of AIDS, with 12.9% stating otherwise. Among those with primary education, 87.3% expressed fear of AIDS, whereas 12.7% did not. Among participants with JHS education, 70.0% reported being afraid, and 30.0% said they were not. The *p* value for the relationship between educational level and fear of AIDS was 0.919.

**TABLE 4:** Proportion of *Kayayei* who report fear of acquired immunodeficiency syndrome (AIDS) by age and educational level.

	Whether *Kayayei* are afraid of AIDS?		
Variable	Yes (%)	No (%)	*p* value
**Age**			0.013
10–12	74 (86.0)	12 (14.0)	
13–15	80 (84.2)	15 (15.8)	
16–17	188 (85.8)	31 (14.2)	
**Education**			0.919
Never been to school	81 (87.1)	12 (12.9)	
Primary	233 (87.3)	34 (12.7)	
JHS	28 (70.0)	12 (30.0)	
**Total**	342 (85.5)	58 (14.5)	

Abbreviation: JHS, junior high school.

### 4.5 Fear and Non‐Fear of AIDS Among *Kayayei*

The narratives gathered through KIIs and FGDs revealed contrasting perspectives among *Kayayei* regarding AIDS. Although some expressed profound fear about contracting the disease, others, though in the minority, voiced reasons for their lack of concern. These opposing views were often shaped by a combination of lived experiences, formal and informal education, cultural and spiritual beliefs, and perceptions of vulnerability and control. Tables 5 and 6 highlight selected quotes that underscore the diverse reasoning behind both fear and non‐fear of AIDS among this population.

**TABLE 5:** Reasons *Kayayei* are afraid of acquired immunodeficiency syndrome (AIDS).

Quote ID	Sample quote
**Q1**	‘I'm scared of AIDS because I think there isn't a cure for it. You die a slow, terrible death once you get it. I'm scared because I've seen people waste away, and I don't want to go through that’. (FGD, 16‐year‐old, Agbogbloshie)
**Q2**	‘Something I know about AIDS is that it will kill you once you have it. We were all afraid of COVID‐19, but at least that could be cured. This fact alone scares me because it seems like AIDS can't be stopped. To avoid that fate, I'm careful about what I do’.(KII, 17‐year‐old, Makola)
**Q3**	‘I've heard that AIDS makes your body weak and skinny, which lets everyone know you're dying. Being ashamed of being sick scares me. I'm afraid of being judged and rejected, so I try to keep myself safe to escape that’. (FGD, 15‐year‐old, Makola)
**Q4**	‘Once people find out you have AIDS, they avoid you. It's too scary to think about being alone and rejected. Because I don't want to lose friends and group support, I'm careful not to get infected’. (KII, 14‐year‐old, Agbogbloshie)
**Q5**	‘When we were in school, we learnt that AIDS can't be cured and is spread by careless behaviour. This information worries me because it seems to say that not being responsible could have bad results. So, I try to stay away from risky sexual activities because I want to keep myself safe from a disease that looks so dangerous’. (FGD, 13‐year‐old girl, Agbogbloshie)

Abbreviations: FGD, focus group discussion; KII, key informant interview.

**TABLE 6:** Reasons *Kayayei* are not afraid of acquired immunodeficiency syndrome (AIDS).

Quote ID	Sample quote
**Q6**	‘I find it difficult to comprehend why AIDS should be a source of fear, as I think it mostly affects individuals who are cursed or those who engage in promiscuous lifestyles, like having numerous partners. I have confidence that my decisions and my beliefs shield me from these kinds of diseases, which gives me a sense of security’. (FGD, 16‐year‐old girl, Agbogbloshie)
**Q7**	‘I believe God protects me as long as I avoid immoral behaviour, hence I do not think I will contract AIDS. My beliefs and careful behaviour protect me against such diseases’. (KII, 15‐year‐old, Agbogbloshie)
**Q8**	‘Not only AIDS can kill, but also malaria, tuberculosis, and recently, COVID‐19. Because death can come from anything, I don't worry about it. Getting my daily bread is my priority, not fear of getting AIDS’. (FGD, 17‐year‐old girl, Makola)
**Q9**	‘Medicines have enabled individuals with AIDS to maintain their health and strength. This is not the same as in the past, when it was seen as a death sentence. This makes me feel better because I know that medicine can help you live a normal life’. (KII, 14‐year‐old, Agbogbloshie)
**Q10**	‘I keep myself safe by using condoms and being careful about who I date. I'm not afraid of getting AIDS because I don't sleep around without considering the implications of being reckless. I'm sure I'll stay safe because I make smart decisions and know how to avoid problems’. (FGD, 13‐year‐old girl, Makola)

Abbreviations: FGD, focus group discussion; KII, key informant interview.

#### 4.5.1 Reasons Why Kayayei Are Afraid of AIDS

The fear of AIDS was strongly voiced by many *Kayayei* who participated in this study. Their fears were not only grounded in the incurable nature of the disease but also in the severe health deterioration, stigma and social rejection that they associated with it.

- **Perception of AIDS as incurable and deadly**: Many participants expressed a fear rooted in the belief that AIDS is a slow and painful death sentence. The absence of a known cure amplified their anxiety about contracting the disease and reinforced cautious behaviour (Quotes Q1 and Q2).
- **Fear of physical deterioration and visible symptoms**: Some *Kayayei* feared the visible bodily effects of AIDS, such as weight loss and weakness. This physical decline, they believed, served as a public marker of the disease, exposing them to shame and **judgment** (Quote Q3).
- **Anticipated stigma and social isolation**: The threat of being abandoned or avoided by friends, peers and even family members was a major source of fear. The potential social rejection for having HIV, even before developing AIDS, was seen as emotionally distressing (Quote Q4).
- **Moral and educational messaging**: School‐based and community education played a role in reinforcing the idea that AIDS was a consequence of irresponsible sexual behaviour. This moral framing caused some to fear being labelled as bad if they ever contracted the virus, thus intensifying their motivation to stay safe (Quote Q5).
- **Internalized responsibility and personal vigilance**: Some participants associated the avoidance of AIDS with personal responsibility. They were fearful not just of the disease itself but also of the consequences of what they considered poor decision‐making, further pushing them to avoid risky behaviours (Quote Q5).

#### 4.5.2 Reasons Why Some Kayayei Are Not Afraid of AIDS

Although the predominant sentiment among the *Kayayei* was a fear of AIDS, a small group articulated reasons for not approaching the disease with the same level of anxiety. The KIIs and FGDs indicated that some *Kayayei* regarded these reasons as reflecting fatalism, religious faith, misinformation and a reliance on modern medicine or personal practices.

- **Attribution of AIDS to immoral lifestyles or curses**: Some participants held the belief that AIDS solely impacts individuals with ‘bad’ lifestyles, such as those who are promiscuous or deemed cursed. This moral distancing enabled them to perceive themselves as personally exempt and, consequently, less vulnerable (Quote Q6).
- **Religious faith as protection**: Religious convictions provided some *Kayayei* with a sense of divine immunity. They believed that by following moral and spiritual guidelines, they would be safeguarded against diseases such as AIDS (Quote Q7).
- **Fatalistic outlook and competing life challenges**: Several participants minimized their fear of AIDS by highlighting that other diseases, such as malaria, COVID‐19 and tuberculosis, also lead to deaths. They believed that concentrating on a single illness was less practical than tackling their ongoing challenges related to daily survival (Quote Q8).
- **Belief in medical advances and treatment availability**: The notion that AIDS is no longer a death sentence due to modern medicine has also diminished fear. Access to antiretroviral therapy is viewed as a means to live a normal life despite being infected (Quote Q9).
- **Confidence in personal prevention strategies**: Some *Kayayei* felt empowered by their own behaviours, such as using condoms and carefully selecting sexual partners. Their confidence in these preventive measures lessened their fear of contracting AIDS (Quote Q10).

## 5 Discussion

This study explored *Kayayei's* perceptions of HIV/AIDS, the influences shaping these views, their levels of fear regarding infection, and the reasons behind their emotional responses. The findings reveal a widespread belief among *Kayayei* that AIDS is a serious and life‐threatening condition, with 83.5% of participants affirming its severity. This perception aligns with existing research from SSA, which consistently underscores the recognition of AIDS as a major public health concern, particularly among socially and economically marginalized groups [51, 52]. From a policy perspective, the high recognition of AIDS as a serious disease indicates that public health messaging has effectively communicated the gravity of HIV infection. However, awareness alone may not ensure the adoption of protective behaviours [22, 23]. This underscores the need for policy interventions that extend beyond awareness campaigns to include targeted behavioural support and accessible prevention services for highly mobile and economically vulnerable populations, such as the *Kayayei*. Within the HBM framework, this pattern reflects a strong perception of disease severity, but it does not necessarily lead to behavioural change when other constructs, such as perceived susceptibility and perceived barriers, are unevenly distributed across the population.

Framed within the HBM, these findings reflect a high degree of perceived severity, one of the model's core constructs. The *Kayayei's* awareness of AIDS as a deadly disease suggests a cognitive acknowledgement of the health risks involved. Although the quantitative analysis did not reveal statistically significant associations between age or education level and perceived severity (*p* = 0.256 and *p* = 0.390, respectively), a notably higher proportion of respondents with JHS education (92.5%) recognized the disease's seriousness. This pattern, while not conclusive, suggests that educational attainment may modestly contribute to heightened health risk awareness, a finding consistent with health literacy literature and its emphasis on education as a facilitator of informed health decisions [53, 54]. These findings emphasize the need to integrate sexual and reproductive health education into formal schooling and community‐based programmes targeting out‐of‐school youth. Policies that expand educational access and provide health literacy initiatives for migrant adolescents can strengthen HIV prevention efforts. From an HBM perspective, improving education may enhance perceived susceptibility and self‐efficacy, helping individuals recognize their vulnerability to infection and their ability to adopt preventive behaviours.

Perceptions of severity were further evident in the specific beliefs articulated by respondents. A substantial proportion indicated that AIDS ‘kills fast’ (39.4%) or ‘has no cure’ (23.8%). These beliefs serve as potent cues to action, reinforcing the salience of AIDS as a health threat and motivating protective behaviours, in line with HBM predictions [38]. The persistence of the belief that AIDS ‘kills fast’ likely reflects historically embedded community narratives from earlier phases of the HIV epidemic, when treatment options were limited, and mortality rates were higher. In many SSA communities, early experiences with AIDS were characterized by visible physical deterioration and death, leading to enduring social memories of the disease as rapidly fatal [55]. This underscores the need for prevention messaging that communicates not only the seriousness of HIV but also provides clear information on treatment availability and the benefits of early testing and adherence to antiretroviral therapy. Such balanced messaging may reduce misconceptions while reinforcing the importance of prevention. Within the HBM framework, these narratives serve as powerful cues to action, shaping perceptions of severity and influencing behavioural decision‐making among vulnerable populations.

In contrast, a minority expressed beliefs indicative of reduced perceived threat or fatalistic attitudes, for instance, that HIV ‘only affects cursed people’ (4.4%) or that ‘awareness campaigns have reduced its danger’ (1.0%). Such views mirror religious or moralistic framing of illness, which has been identified in other contexts as diminishing perceived susceptibility and impeding risk‐avoidant behaviours [56]. These findings highlight the importance of culturally responsive HIV interventions that involve religious leaders, community influencers and peer educators in shaping health communication strategies. Policies that incorporate community‐based engagement approaches are likely to be more effective in addressing deeply embedded cultural interpretations of illness and risk. Within the HBM framework, these beliefs demonstrate how low perceived susceptibility can weaken the motivation to adopt preventive behaviours, even when individuals recognize the general severity of the disease.

Fear of AIDS was pervasive, with 85.5% of respondents expressing concern. Interestingly, the highest fear levels were observed among younger *Kayayei* aged 10–12 (86.0%), suggesting that emotional responses to perceived health threats may be especially acute during early adolescence. According to HBM, such emotional reactions, particularly fear, can act as strong motivators for behaviour change [30]. Education level, however, exhibited a more nuanced relationship with fear. Although both uneducated and primary‐educated *Kayayei* reported similarly high fear levels (87%), only 70% of JHS‐educated respondents reported fear. This inverse relationship may reflect increased self‐efficacy, another key HBM construct, among more educated participants. Greater educational attainment may enhance not only health knowledge but also confidence in one's ability to prevent infection, thereby mitigating fear [26]. These findings suggest that education can simultaneously enhance awareness and reduce emotional distress by empowering individuals to take preventive action. From a policy perspective, interventions that strengthen self‐efficacy, such as peer education, youth‐friendly health services and access to condoms and HIV testing, may be particularly effective in translating awareness into sustained preventive behaviour.

Qualitative narratives substantiate and elaborate upon these quantitative patterns. Participants who expressed fear often cited the incurable nature of HIV/AIDS, the stigma associated with the illness, and its visible physical consequences. These insights highlight how perceived severity and perceived barriers interact, reinforcing both the seriousness of the disease and the social implications of infection. Importantly, these fears appeared to catalyse avoidant behaviours, aligning with prior research on the motivational role of fear in public health contexts [57]. Conversely, respondents who did not fear the disease often grounded their sense of security in religious beliefs (God will protect me), advances in medical treatment (e.g., antiretroviral therapy), moral confidence, or the prioritization of other pressing health threats. This finding particularly illustrates the interaction between individual health perceptions and structural constraints. For many *Kayayei* living in economic precarity, immediate survival needs, such as securing daily income and food, often take precedence over long‐term health risks. This tension underscores a key limitation of the HBM in contexts of extreme poverty: Even when individuals recognize the severity of a disease, structural pressures may restrict their ability to act on that knowledge.

The contrasting perceptions observed among participants suggest uneven awareness of HIV treatment and survival outcomes. Although some *Kayayei* recognized that individuals living with HIV can remain healthy through antiretroviral therapy, others continued to associate the disease primarily with death and severe suffering. Similar disparities in treatment knowledge have been documented among socially marginalized and mobile populations in Ghana and other parts of Africa, often linked to inconsistent access to health information and services ([32, 58]). These patterns underscore the need to strengthen outreach‐based HIV education programmes within market spaces and informal settlements where *Kayayei* live and work.

It is also important to consider the sociocultural diversity within the *Kayayei* population. Although most migrant head porters originate from the northern regions of Ghana, they come from several distinct ethnic groups, including Dagomba, Mamprusi, Gonja, Frafra and others, which differ in language, traditions and cultural interpretations of illness and morality [31, 37]. Although these groups share certain regional experiences and migration patterns, they are not culturally homogeneous [9, 10]. Variations in religious practices, community norms, and access to education may, therefore, shape how individuals interpret HIV risk, treatment and prevention messages [23, 35]. For policymakers, this diversity underscores the need for culturally sensitive health communication strategies that recognize linguistic and cultural differences within migrant populations.

When situated within the broader literature, the findings reflect patterns seen in other Ghanaian contexts, where risk perception and health beliefs are associated with the adoption of preventive behaviours [59, 60]. However, this study also uncovers evidence of fatalism and stigmatizing beliefs, echoing findings from South African and Nigerian adolescents, where cultural and religious ideologies have been shown to mediate perceptions of HIV risk and influence behavioural outcomes 61, 62. These parallels underscore the importance of accounting for sociocultural context when designing health interventions, especially for mobile and marginalized populations like the *Kayayei*.

The HBM framework has proven useful in capturing the cognitive and affective dimensions of *Kayayei's* beliefs about HIV/AIDS, highlighting how perceived severity, susceptibility, self‐efficacy and cues to action intersect to shape their attitudes and potential health behaviours. Nevertheless, the model's emphasis on individual‐level determinants must be complemented by an understanding of the structural constraints faced by this population. Extreme poverty, housing insecurity, limited access to health services and social exclusion may inhibit the translation of risk perception into actual behavioural change. These barriers suggest that interventions must extend beyond educational messaging to address broader social determinants of health [63]. Policy responses should, therefore, combine behavioural interventions with structural support measures, including improved access to youth‐friendly health services, safe accommodation programmes for migrant girls and social protection initiatives targeting vulnerable urban migrants.

In conclusion, although the HBM offers a robust lens for understanding AIDS risk perception among *Kayayei*, meaningful change will require integrated strategies that combine individual‐level education with structural interventions. Programmes that strengthen self‐efficacy, provide access to services and engage with cultural belief systems will be more effective in reducing vulnerability and promoting safer health behaviours among internally displaced and economically marginalized groups in Ghana.

## 6 Limitations and Strengths

This study applied the HBM as a theoretical lens and employed a concurrent mixed‐methods design to investigate perceptions and fears of AIDS among *Kayayei* in two Ghanaian markets. Although the HBM provided a robust framework to analyse how perceived severity, susceptibility, self‐efficacy and cues to action shape *Kayayei's* attitudes toward HIV, it primarily focuses on individual cognitive processes and may not fully account for structural determinants like poverty, housing insecurity or gender‐based power dynamics influencing behaviour. Thus, although the HBM enriched the interpretative depth, it should be complemented with structural or ecological models in future research.

Methodologically, the use of both survey and qualitative interviews allowed for data triangulation and a richer understanding of *Kayayei*’s perceptions. However, reliance on self‐reported data may have introduced recall or social desirability bias. To mitigate these potential biases, the study employed several strategies, including the use of trained female research assistants familiar with the market environments, the assurance of confidentiality and anonymity during data collection and the use of multiple data sources (surveys, FGDs, and KIIs) to triangulate responses. These measures were intended to encourage honest responses and enhance the credibility of the findings.

Furthermore, purposive sampling in two urban markets limits generalizability to all *Kayayei* in Ghana. This limitation was partially addressed by selecting Makola and Agbogbloshie markets, two of the largest commercial hubs in Accra with significant concentrations of migrant head porters from different northern regions of Ghana. The inclusion of participants from diverse ethnic and linguistic backgrounds within these markets helped capture a broader range of experiences and perspectives among *Kayayei*. Despite these limitations, the study's integration of quantitative and qualitative insights, context‐specific application of theory and focus on an underserved population present significant strength, offering practical and policy‐relevant implications for HIV prevention interventions among mobile, high‐risk groups.

Another strength of the study lies in its focus on *Kayayei*, a population that remains under‐represented in HIV research despite their socio‐economic vulnerability and mobility. By combining statistical analysis with qualitative narratives, the study provides nuanced insights into both the cognitive and sociocultural dimensions of HIV risk perception among migrant adolescent girls and young women in urban Ghana.

## 7 Conclusion and Implications for Policy and Practice

This study shows that the vast majority of *Kayayei* in Makola and Agbogbloshie perceive AIDS as dangerous and are afraid of the disease, influenced by a combination of high perceived severity and susceptibility (Figure 1). Strong emotional responses, especially fear, among younger and less educated *Kayayei* reveal potential readiness for preventive behaviour. However, recurrent fatalistic, religious or medicalized narratives among those less afraid suggest gaps in perceived susceptibility and efficacy.

To strengthen HIV prevention efforts, programmes should leverage these insights by reinforcing the severity of AIDS and addressing modifiable constructs: perceived susceptibility (emphasizing that anyone can get infected), benefits of prevention and self‐efficacy (building confidence in protective behaviours). Interventions could include personalized risk assessments, peer‐led storytelling to resonate culturally and practical condom skills training to foster empowerment.

Policy interventions must also remove structural barriers. Regular mobile testing and condom distribution in market centres where *Kayayei* work, especially Agbogbloshie, would reduce access constraints. Partnerships with community leaders and faith‐based groups could help counter moralistic or fatalistic beliefs by aligning HIV messages with cultural values. Additionally, integrating mental health and stigma‐reduction modules into HIV education would address emotional and social barriers, enhance self‐efficacy and reduce fear‐based avoidance behaviours.

Beyond individual‐level interventions, policy responses should also recognize the broader social and economic vulnerabilities shaping the lives of *Kayayei*. Many migrant head porters live in informal settlements near market areas with limited access to healthcare services, sanitation and stable housing. Strengthening outreach‐based sexual and reproductive health services in market environments, such as youth‐friendly clinics, mobile health units and peer health educators, could improve access to testing, counselling and prevention resources for this population.

Furthermore, because *Kayayei* originate from several culturally distinct communities across northern Ghana, culturally responsive communication strategies that incorporate local languages, community norms and religious values are likely to be more effective than generic health campaigns. Engaging market associations, community leaders and faith‐based organizations in HIV education initiatives may therefore enhance trust and improve the relevance of prevention messaging.

By applying the HBM in combination with structural support strategies, policymakers and practitioners can design interventions that are context‐sensitive, responsive to *Kayayei*’s lived realities and capable of closing the gap between awareness and behaviour. This evidence‐based approach could serve as a blueprint for similar high‐risk, mobile populations across SSA.

## Author Contributions

**Sylvester Kyei‐Gyamfi**: conceptualization, investigation, methodology, validation, software, formal analysis, data curation, supervision, resources, project administration, visualization, writing – review and editing, writing – original draft.

## Funding

The author has nothing to report.

## Ethics Statement

Ethical approval for this study was obtained from the National Child Protection Committee (NCPC) in February 2020. The research was conducted in full accordance with relevant ethical guidelines for research involving human participants, including the principles outlined in the Declaration of Helsinki.

## Consent

Written and/or verbal informed consent was obtained from all participants prior to data collection. Participants were informed of the voluntary nature of the study, their right to withdraw at any stage and the option to decline answering any questions. All data were anonymized to safeguard participant privacy and confidentiality.

## Conflicts of Interest

The author declares no conflicts of interest.

## Data Availability

Due to ethical considerations and the need to protect participant confidentiality, the data supporting this study are not publicly available. However, data may be made available upon reasonable request to the corresponding author, subject to institutional and ethical approval.
